# Embryonic Stem Cell-Derived Factors Inhibit T Effector Activation and Induce T Regulatory Cells by Suppressing PKC-θ Activation

**DOI:** 10.1371/journal.pone.0032420

**Published:** 2012-03-07

**Authors:** Kanishka Mohib, Bodour AlKhamees, Haggag S. Zein, David Allan, Lisheng Wang

**Affiliations:** 1 Department of Biochemistry, Microbiology and Immunology, Faculty of Medicine, University of Ottawa, Ottawa, Ontario, Canada; 2 Regenerative Medicine Program, Ottawa Health Research Institute, University of Ottawa, Canada; 3 Ottawa Institute of Systems Biology, University of Ottawa, Ottawa, Ontario, Canada; Ohio State University Medical Center, United States of America

## Abstract

Embryonic stem cells (ESCs) possess immune privileged properties and have the capacity to modulate immune activation. However, the mechanisms by which ESCs inhibit immune activation remain mostly unknown. We have previously shown that ESC-derived factors block dendritic cell maturation, thereby indirectly affecting T cell activation. Here, we show that ESC-derived factors also directly affect T cell activation. We provide the first demonstration that ESC-derived factors significantly down-regulated the expressions of IL-2 and IFN-γ, while markedly up-regulating the expression of IL-10, TGF-β, and Treg transcription factor Foxp3 in CD4+ CD25+ T cells. Furthermore, ESC-derived factors robustly suppressed T cell proliferation in response to the protein kinase C-θ (PKC-θ) activator phorbol 12-myristate 13-acetate (PMA). Western blot analysis indicated that ESC-derived factors prevented PKC-θ phosphorylation without influencing total PKC-θ levels. Moreover, IκB-α degradation was abrogated, confirming absence of PKC-θ activity. The impact of ESC-derived factors on PKC-θ activation appeared to be specific since other upstream T cell signaling components were not affected. In conclusion, ESCs appear to directly impact T cell activation and polarization by negatively regulating the PKC-θ pathway.

## Introduction

Embryonic stem cells (ESCs) are pluripotent stem cells and are able to differentiate into cells derived from all three germ layers [1], [2], [3]. As such, they represent an important tool for the study of developmental biology and may provide new treatment for a variety of degenerative and genetic diseases [4], [5], [6], [7], [8], [9].

Recently, several groups have described that ESCs possess immune privileged properties. These properties allow ESCs to survive across both allogeneic and xenogeneic barriers without evoking immune responses [10], [11], [12], [13], [14], [15], [16], [17], [18], [19]. The ability of ESCs to evade the immune system may be associated with their very low level of MHC I expression and no MHC II expression [10], [11], [12], [14], [15], [16]. In addition, ESCs lack expression of co-stimulatory molecules CD80, CD86 and CD40 that may contribute to activating immune effector cells [10], [11], [12], [14], [15], [16]. However, differentiation of ESCs and treatment with inflammatory cytokines such as IFN-γ results in MHC I expression and immune recognition [11], [15]. Notably, these properties are found to be consistent in human, mouse and rat ESCs [11], [12], [16].

In addition to evading the immune system, ESCs also have the capacity to actively modulate the immune system towards a tolerant state. In mixed lymphocyte reaction assays, ESCs suppress immune activation and proliferation in response to third party antigen presenting cells (APCs) [14], [15]. It has become apparent that ESCs are able to influence APCs [11], [14], [15], [20]. Other studies have elucidated that ESCs are able to directly inhibit T cell and NK cell activity [11], [21], [22]. Significantly, rat ESCs were shown to provide immune protection to solid organ transplants across allogeneic barrier [12]. Therefore, ESCs possess powerful immune modulatory properties that not only facilitate their own survival in hostile immunological environments but also inhibit immune responses to third party APCs and provide protection to solid organ transplants.

Harnessing these immune modulatory properties may yield important applications in autoimmune conditions, allergy and transplantation. However, ectopic tumor (teratoma) formation after using live ESCs in vivo is the most serious safety concern [1], [2], [3]. This has caused great apprehension in recent clinical trials when transplanted embryonic neuronal precursors gave rise to spinal cord and brain stem tumors [23]. As a result, use of live ESCs to promote immune tolerance and reduce the severity of aberrant or unwanted immune activation is currently limited by potential serious adverse effects [2], [24], [25], [26], [27]. Therefore, alternative strategies that can circumvent tumor formation while retaining the immune modulatory properties of ESCs are needed.

Recently, we have established that cytoplasmic lysates of both human and mouse ESCs retain the immune modulatory properties of live cells thereby averting the potential of teratoma formation. Our studies elucidated that these ESC-derived factors have the capacity to inhibit maturation of monocyte-derived DCs [20]. ESC-derived factors prevented full maturation of DCs in response to TNF-α by decreasing surface expression of CD80, MHC II and CD83 molecules. Accordingly, DCs treated with ESC-derived factors retained greater phagocytic ability, secreted low levels of IL-12p40 and were poor stimulators of allogeneic T cells [20]. Interestingly, we observed that inhibition of T cell activation by DCs could be enhanced further by addition of ESC-derived factors during the T cell activation assay, suggesting that ESC-derived factors may also affect T cell activation.

Here we show that ESC-derived factors have the capacity to modulate T cell function directly. ESC-derived factors suppress the upregulation of T cell activation markers CD25, CD44, and CD69. They also inhibit IFN-γ production in T cells while promoting Foxp3 expression. In addition, we provide the first evidence that ESCs suppress PKC-θ activation without affecting upstream signaling components originating from CD3 and CD28 receptors. Moreover, ESC-derived factors work synergistically with very low dose of immunosuppressive drug cyclosporine (calcineurin inhibitor) to markedly suppress T cell proliferation in response to allo-antigen. Hence, ESC-derived factors may hold the potential to be used as a therapeutic, instead of live ESCs, in overcoming aberrant immune responses.

## Results

### ESC-derived factors directly inhibit T cell proliferation and activation

**Figure 1:**
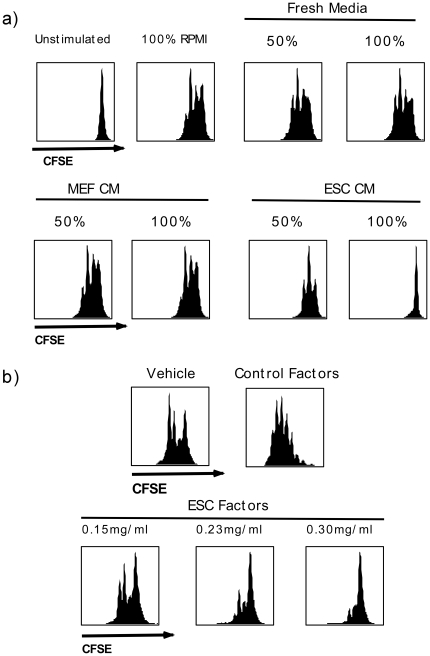
ESC-conditioned media and cellular factors from ESC-extracts inhibit T cell proliferation in response to anti-CD3/anti-CD28 stimulation. *a)* C57BL/6 splenocytes were labeled with CFSE and activated with anti-CD3/anti-CD28 in RPMI media (unstimulated cells presented in panel 1 and stimulated cells in panel 2). Cells were also activated in 50% RPMI along with 50% or 100% fresh unconditioned media (panels 3 and 4), 50% RPMI along with 50% or 100% mouse embryonic fibroblast-conditioned media (MEF-CM, panels 5 and 6), and 50% RPMI along with 50% or 100% mouse ESC-conditioned medium (ESC-CM, panels 7 and 8). After 48 hours the cells were analyzed by flow cytometry for proliferation. *b)* ESCs were grown in feeder free cultures, harvested and lysed by sonication. Cell membrane, mitochondria and nucleus were removed by centrifuging the sonicate at 15000 g for 15 minutes. Proliferation of B6 splenocytes stimulated with anti-CD3/anti-CD28 in RPMI media was assessed using extraction buffer alone (vehicle, panel 1), lysates from C2C12 cells (Control-Factors) or increasing concentration of ESC-derived factors (ESC-Factors). Results are representative of 4 separate experiments.

In order to specifically determine the impact of ESCs on T cells, we stimulated mouse B6 splenocytes with anti-CD3 and anti-CD28 in the presence of increasing concentrations of ESC-conditioned media (ESC-CM) or control MEF cell-conditioned media. We found that ESC-CM inhibited T cell proliferation as indicated by reduced number of cell divisions reflected by lower degree of CFSE dilution (Figure 1a). Moreover, increasing amounts of ESC-CM (from 50% in combination with RPMI media to 100% of ESC-CM) had a greater inhibitory effect on T cell proliferation (Figure 1a). In contrast, non-conditioned media and media conditioned by MEF cells did not have an effect on anti-CD3 and anti-CD28 mediated T cell proliferation (Figure 1a). To further confirm that T cell inhibition was due to ESC-derived factors, we prepared and examined the cytoplasmic fraction of ESCs (see materials and methods) on T cell proliferation. Splenocytes were stimulated with anti-CD3 and anti-CD28 in the presence of increasing concentrations of ESC-derived factors. Similar to ESC-CM, ESC-derived factors inhibited T cell proliferation in a dose dependent manner (Figure 1b). The inhibitory effect of ESC-derived factors was also examined in mixed lymphocyte reaction (MLR) assays. Once again, ESC-derived factors were able to inhibit T cells proliferation in a dose dependent manner (Figure S1). Conversely, vehicle control did not affect T cell proliferation, while mouse muscle stem cell precursor-derived factors (C2C12 cell line) enhanced T cell proliferation (Figure 1b). Notably, human ESC-derived factors from H9 cell line [11], [14], [15], [20] were also found to inhibit T cell proliferation in response to concanavalin A and PMA (Figure S2).

**Figure 2:**
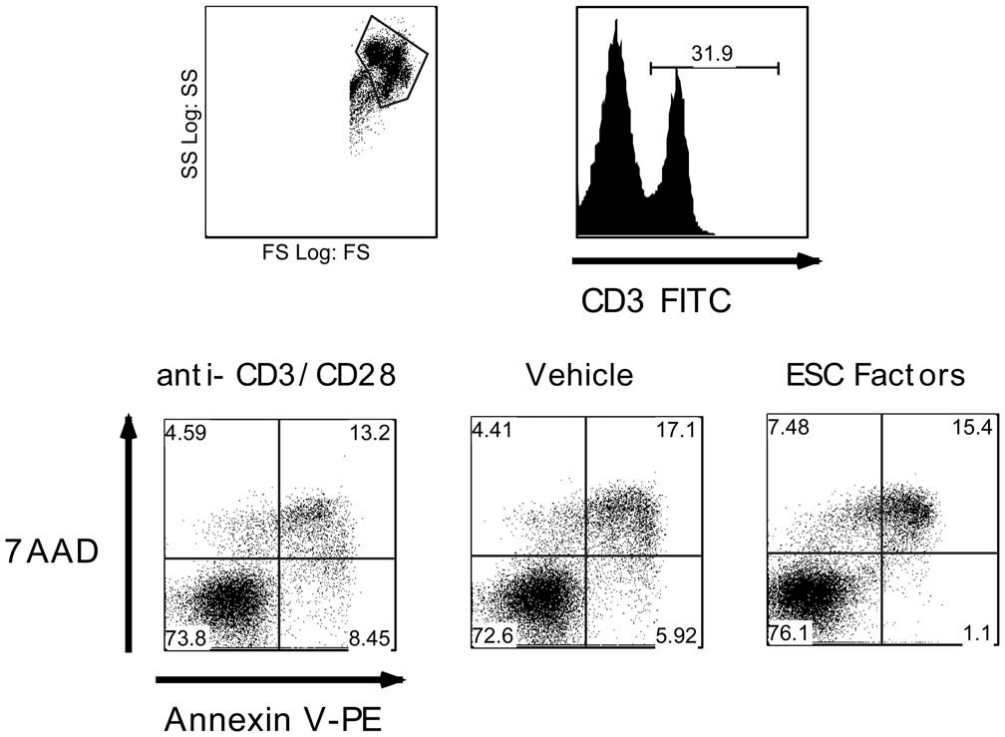
ESC-derived factors do not enhance T cell death. C57BL/6 splenocytes were stimulated with anti-CD3/anti-CD28 antibodies in the presence of ESC-derived factors for 24 hours. The cells were harvested and washed with PBS and stained with anti-CD3 antibody, Annexin V-PE and 7AAD to examine T cell apoptosis and necrosis, respectively. Analysis was carried out by gating on CD3+ cells followed by determination of Annexin V-PE and 7AAD. Results are representative of 4 separate experiments.

To elucidate whether T cell inhibition is due to activation-induced cell death (AICD) by ESC-derived factors, we examined T cell apoptosis and necrosis using Annexin V and 7AAD respectively. Splenocytes stimulated with anti-CD3 and anti-CD28 and treated with ESC-derived factors were found to have the same number of CD3+ dead cells as control treatments 24 hours following stimulation (Figure 2). Moreover, the number of dead T cells did not significantly increase after stimulation with anti-CD3 and anti-CD28 and treatment with ESC-derived factors for 72 hours (Figure S3). These data suggests that ESC-derived factors inhibit T cell proliferation without inducing T cell death following activation.

**Figure 3:**
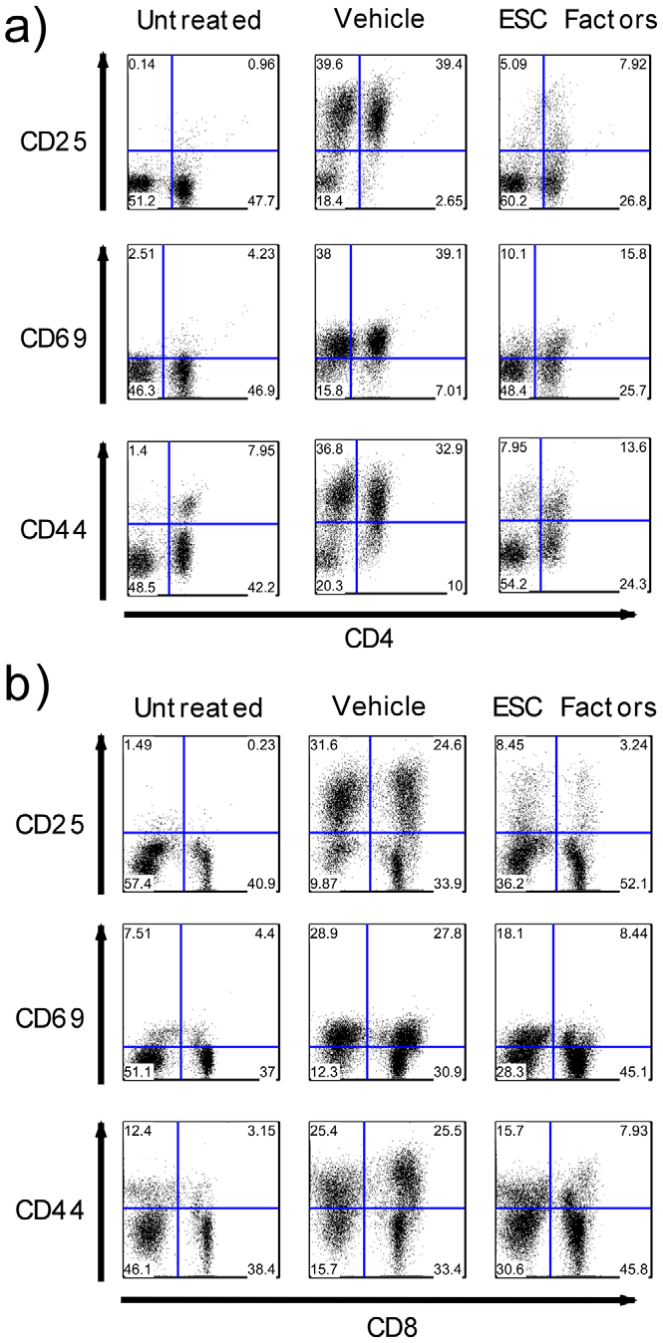
ESC-derived factors inhibit upregulation of activation markers CD25, CD44 and CD69 on CD4 and CD8 T cells. Negatively isolated C57BL/6 CD3+ T cells were stimulated with plate bound anti-CD3/anti-CD28 antibodies in the presence of ESC-derived factors or vehicle control. *a)* CD4 positive T cells were examined for CD25 and CD44 expression after 24 hours and CD69 expression after 6 hours of stimulation. *b)* CD8 positive T cells were examined for CD25 and CD44 expression after 24 hours and CD69 expression after 6 hours of stimulation. Results are representative of 3 separate experiments.

Next we assessed the impact of ESC-derived factors on T cell activation. CD3+ T cells were negatively selected from splenocytes and stimulated with anti-CD3 and anti-CD28. Subsequently, T cells were examined at the specified time points for the surface expression of CD25, CD44 and CD69. These markers are important for T cell activation and subsequent proliferation and function [28], [29], [30]. We found that ESC-derived factors have the capacity to markedly decrease the surface expression of CD25 on both CD4 and CD8 T cells (Figure 3a,b). Similarly, ESC-derived factors were also able to noticeably reduce the surface expression of CD44 and CD69 (Figure 3a.b). As a result, it can be concluded that ESC-derived factors inhibit T cell proliferation by decreasing surface expression of important markers that are necessary for proper activation and subsequent proliferation.

### ESC-derived factors skew T cell cytokine production towards a T regulatory profile

**Figure 4:**
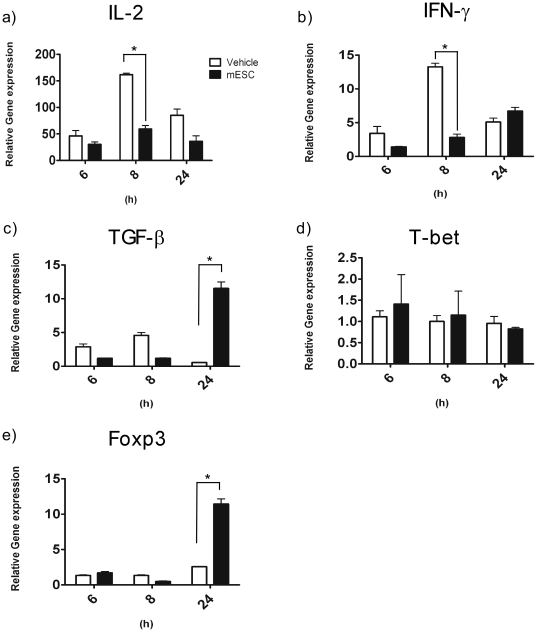
ESC-derived factors modulate T helper responses during an allogeneic immune response. A one way mixed lymphocyte reaction was performed, C57BL/6 splenocytes were used as responders and CD1 splenocytes as stimulators in the presence of ESC-derived factors or vehicle control. Cells were harvested at the indicated time points and total RNA was isolated. Subsequently, cDNA was synthesized and used to carry out QPCR to examine the expression of cytokines and master regulator transcription factors of T helper cells. *a)* IL-2, *b)* IFN-γ, *c)* TGF-β, *d)* T-bet, *e)* Foxp3. Responders alone were used as baseline. Results are representative of 3 separate experiments. Data points represent mean ± SD. * indicates p value≤0.05. White bars represent results obtained from vehicle treated MLRs and black bars indicate results obtained from ESC-derived factor treated MLRs.

The ability of ESC-derived factors to affect proper T cell activation led us to examine T cell effector function in response to alloantigen. We performed one-way mixed lymphocyte reaction (MLR) assays and examined the expression of various cytokines and transcription factors by quantitative PCR at several time points. We found that MLR treated with ESC-derived factors had significantly lower expression levels of IL-2 and IFN-γ after 8 hours of stimulation compared to controls (Figure 4 a,b). In contrast, we observed significantly higher expression of TGF-β and Foxp3 in the same samples by 24 hours (Figure 4c,e). However, Tbet expression remained unchanged (Figure 4d). These results indicate that ESC-derived factors may favour development of T regulatory cells over Th1 cells in response to alloantigen.

**Figure 5:**
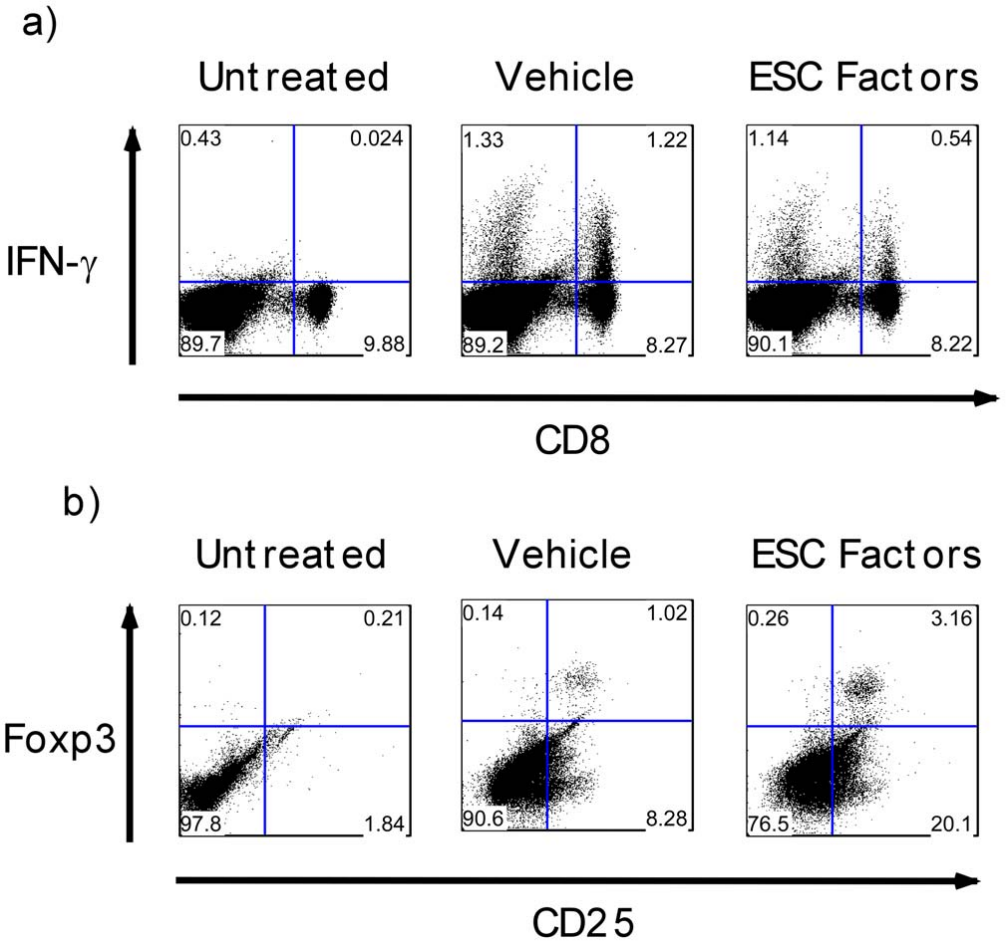
ESC-derived factors skew T cell helper responses towards T regulatory cells. *a)* C57BL/6 splenocytes were pre-treated over night with ESC-derived factors and stimulated with PMA and Ionomycin or anti-CD3/anti-CD28 for 6 hours. Protein transport inhibitor cocktail was added to the cells 1 hour following stimulation. Cells were harvested and stained for surface CD8. After washing, the cells were fixed, permeabilized and stained for intracellular IFN-γ. *b)* C57BL/6 splenocytes were treated with ESC-derived factors and stimulated with anti-CD3 and anti-CD28 for 3 days. The cells were harvested and stained for CD3, CD4 and CD25. Subsequently, cells were fixed, permeabilized and stained for Foxp3. Gates were set on CD3+ followed by CD4+ cells. Results are representative of at least 3 separate experiments.

To further confirm that the shift in cytokine and transcription factors was specifically induced in T helper subsets, we examined IFN-γ and Foxp3 expression by intracellular staining of T cell subsets. Splenocytes were stimulated with anti-CD3 and anti-CD28 or PMA/Ionomycin in the presence of ESC-derived factors or controls and subsequently analyzed by flow cytometry at the indicated time points. We gated on CD4 and CD8 T cell subsets and found that ESC-derived factors suppressed IFN-γ production in CD8 T cells compared to controls (Figure 5a) but not in CD4 cells at this time point (data not shown). Next we examined the frequency of T regulatory cells based on the combined expression of CD4, CD25 and Foxp3 [31]. Treatment of splenocytes with ESC-derived factors resulted in greater number of CD4+, CD25+ and Foxp3+ cells compared to controls (Figure 5b). In addition to stimulating splenocytes with anti-CD3 and anti-CD28 or PMA, we also used one-way MLR to examine the intracellular protein levels of IFN-γ and Foxp3. We found that ESC-derived factors decreased the number of IFN-γ-producing cells and increased the number of Foxp3+ cells in response to alloantigen (Figure S4). Taken together, these data suggest that ESC-derived factors may skew the production of cytokine and transcription factors in T cells and promote the development of T regulatory cells.

### ESC-derived factors skew T cells responses by preventing PKC-θ phosphorylation

**Figure 6:**
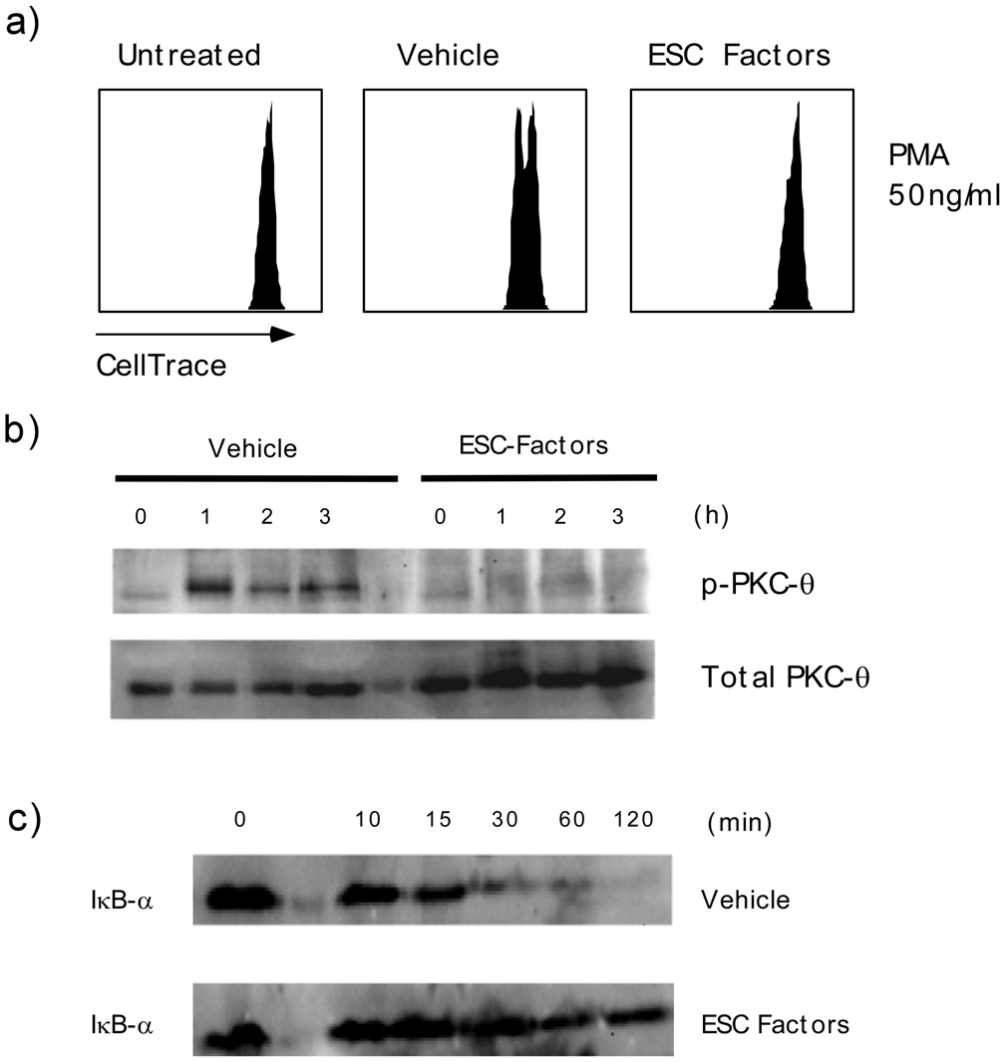
ESC-derived factors inhibit PMA mediated PKC-θ activation in T cells. *a)* C57BL/6 splenocytes were stained with CellTrace Violet Cell Proliferation dye and activated with 50 ng/ml of PMA for 24 hours in the presence of 0.23 mg/ml of ESC- derived factors (without pre-treatment). Cells were harvested, washed with PBS and examined for proliferation by flow cytometry. *b)* C57BL/6 splenocytes were pre-treated over night with 0.23 mg/ml of ESC-derived factors and stimulated with 50 ng/ml of PMA for the indicated periods of time. Subsequently, the cells were harvested and lysed. Lysates were examined by western blotting for PKC-θ phosphorylation (Thre 538), and total PKC-θ. *c)* C57BL/6 CD3+ T cells were pre-treated overnight with 0.23 mg/ml ESC-derived factors and stimulated with 50 ng/ml of PMA for the indicated periods of time. Subsequently, the cells were harvested and lysed. Lysates were examined by western blotting for IκB-α degradation. Results are representative of 4 separate experiments.

The mechanisms underlying ESC-mediated T cell inhibition remain poorly understood. Recently, Yachimovich-Cohen et al. described that intact human ESCs are able to directly inhibit T cell proliferation through the expression of the enzyme arginase-1 [22]. However, in our assays we supplement the cytoplasmic lysates with excess amounts of L-arginine to prevent protein-protein aggregation and dimerization [32], [33]. It is very likely that ESCs may have multiple properties that affect T cell activation and proliferation. To delineate alternate mechanisms, we stimulated splenocytes with various mitogens that activate specific signaling pathways in T cells. Interestingly, we found that ESC-derived factors could specifically inhibit phorbol-1-3-merystate (PMA) mediated proliferation of splenocytes (Figure 6a). Similarly, human ESC-derived factors were able to inhibit PMA-mediated proliferation of healthy donor T cells (Figure S2). It is well known that PMA is a specific activator of protein kinase C-theta (PKC-θ) [34], [35] Therefore, ESC-derived factors may inhibit proper T cell activation and proliferation by suppressing PKC-θ activation.

To directly establish the impact of ESCs on PKC-θ, splenocytes were stimulated with PMA in the presence and absence of ESC-derived factors. Subsequently, western blot assays were carried out to assess PKC-θ phosphorylation at Threonine-538 (Thre-538) that has been shown to be reflective of its activity [36]. Splenocytes treated with ESC-derived factors at various time points had very little or no phosphorylation of PKC-θ whereas control splenocytes showed strong phosporylation in response to PMA (Figure 6b). Notably, ESC-derived factors did not have an effect on total PKC-θ levels as they remained constant at all time points similar to controls (Figure 6b). To determine whether the absence of phosphorylation at Thr-538 was indeed reflective of PKC-θ activity we examined down stream targets of the kinase. PKC-θ is known to form a complex with CARMA-1, Bcl-10 and MALT-1 to induce NFκB translocation to the nucleus by causing degradation of IκB [37]. As a result we examined IκB-α degradation in purified T cells that had been activated with PMA. Consistent with above results, we found that IκB-α was not or only slightly degraded in cells that were treated with ESC-derived factors whereas IκB-α was visibly degraded in controls (Figure 6c). Accordingly, cells treated with ESC-derived factors had decreased levels of NFκB translocated to the nucleus in comparison to those treated with vehicle (Figure S5). Hence, ESC-derived factors can directly affect T cell activation and proliferation by inhibiting PKC-θ phosphorylation and subsequent activity.

### ESC-derived factors specifically inhibit PKC-θ activation without affecting upstream T cell signaling

**Figure 7:**
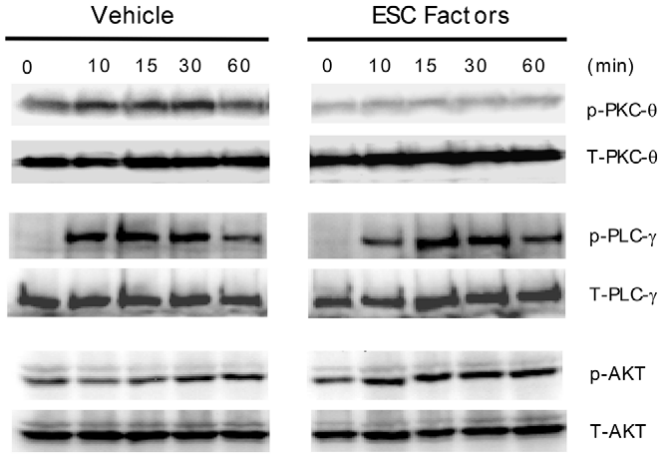
ESC-derived factors specifically inhibit PKC-θ activation without affecting upstream signaling molecules. C57BL/6 splenocytes were pre-treated with ESC-derived factors overnight and stimulated with anti-CD3/anti-CD28 for the indicated periods of time. Subsequently, the cells were harvested and lysed. Lysates were examined by western blotting for PLC-γ, AKT and PKC-θ phosphorylation. Results are representative of 3 separate experiments.

Although ESC-derived factors have the capacity to inhibit PMA mediated PKC-θ activation, it is unclear whether they also affect other signaling components emanating from the TCR and CD28. It is known that PKC-θ activation is mediated by signaling molecules PLC-γ and PI3K originating from the TCR and CD28, respectively [38], [39]. As such, we determined whether these signaling molecules were affected by ESC-derived factors following anti-CD3 and anti-CD28 stimulation. Splenocytes were pre-treated overnight with ESC-derived factors and stimulated with anti-CD3 and anti-CD28 for the indicated time periods. Subsequently, phosphorylation of PLC-γ, AKT (used as a surrogate marker for PI3K) and PKC-θ were examined. Consistent with the data obtained with PMA (Figure 6), we found that splenocytes treated with ESC-derived factors and activated with anti-CD3 and anti-CD28 had little or no phosphorylation of PKC-θ at all time points, whereas phosphorylation was detected in controls (Figure 7). In contrast, phosphorylation of PLC-γ and AKT could be detected in both ESC-derived factor- and control-treated splenocytes at all time points (Figure 7). Therefore, it can be concluded that ESC-derived factors specifically inhibit PKC-θ without influencing its known up-stream activators.

### ESC-derived factors enhance inhibition of allogeneic immune activation in combination with cyclosporin A

**Figure 8:**
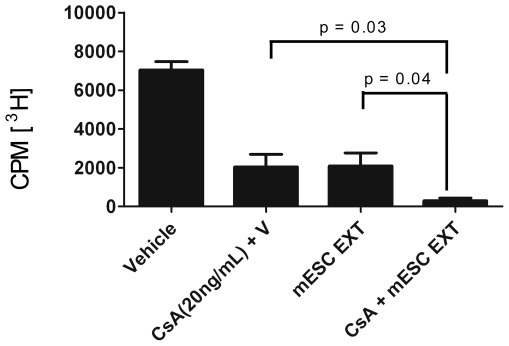
ESC-derived factors in combination with cyclosporin A enhance inhibition of allogeneic immune activation. A one way mixed lymphocyte reaction was performed, C57BL/6 splenocytes were used as responders and CD1 splenocytes as stimulators in the presence of vehicle control, ESC-derived factors and CsA. Moreover, ESC-derived factors were used in combination with CsA to determine whether they can complement one another in preventing allo-immune activation.

By determining that ESC-derived factor specifically inhibit PKC-θ phosphorylation (Figure 6, 7), we then asked whether T cell proliferation could be further suppressed in combination with inhibitors of the calcium-signaling pathway. The effect of ESC-derived factors was tested in combination with CsA, a drug that specifically inhibits calcineurin activation and has been widely used in the clinic to alleviate allogeneic organ rejection. Treatment of MLR with CsA alone resulted in a decrease in cell proliferation (Figure 8). Notably, ESC-derived factors used in combination with CsA markedly suppressed MLR proliferation and significantly enhanced the effect of very low dose CsA drug (Figure 8). These data indicate that ESC-derived factors in combination with inhibitors of the calcium-signaling pathway have an additive effect in preventing allo-immune activation. It can be concluded that ESC-derived factors may hold the potential as a new supplement of current conventional immunosuppressive drugs to promote allogeneic graft survival while reducing harmful side effects.

## Discussion

Several groups have demonstrated that ESCs have immune modulatory properties that not only allow ESCs to survive across allogeneic immune barrier but also provide protection to solid organ transplants [5], [10], [12], [13], [14], [15], [17], [18], [19], [20], [22], [40], [41]. Here we have further demonstrated that ESCs have the capacity to directly inhibit T cell proliferation and activation. Moreover, we have demonstrated for the first time that one of the mechanisms by which ESCs modulate the immune system occurs through suppression of PKC-θ phosphorylation. In addition, we have found that ESC-derived factors skew T helper responses from Th1 towards a regulatory T cell phenotype by decreasing the production of IL-2 and IFN-γ and increasing production of TGF-beta and Foxp3. We also determined that ESC-derived factors were able to specifically inhibit PKC-θ phosphorylation without affecting upstream T cell signaling components derived from both CD3 and CD28, such as PLC-γ and PI3K (known activators of PKC-θ).

The role of PKC-θ and its contribution to T cell function and phenotype has been expanded by several important findings. Zanin-Zhorov's et al. have recently elucidated that PKC-θ is recruited much less in T regulatory cells compared to effecter T cells [42]. They established that PKC-θ has a negative impact on T regulatory cells rendering them less effective in suppressing effecter T cell function. Moreover, inhibition of PKC-θ was shown to enhance T regulatory potency in suppressing effecter T cell proliferation and IFN-γ secretion [42]. Meanwhile, Valenzuela et al. have found that PKC-θ is required for allo-antigen responses in a GVHD model [43]. Whereas wild type T cells induced severe GVHD resulting in a lethal outcome for all mice, PKC-θ-KO T cells induced very mild or no GVHD leading to the survival of most transplanted mice [43]. Finally, several groups have established that in the absence of PKC-θ activation, an anergic genetic program is initiated. This anergic state is induced due to prolonged calcium flux leading to persistent activation and translocation of NFAT to the nucleus [44], [45], [46], [47] and an enhanced expression of E3 ubiquitin ligases that negatively regulate T cell signaling [48], [49], in the absence of other transcription factor such as NFκB and AP-1. Our current results expand on the previous findings and provide the first connection between ESC immune modulation and PKC-θ inhibition. We believe that the inhibition of PKC-θ by ESC-derived factors will have a major impact in modulating T cell responses.

The mechanisms underlying ESC-mediated immune modulation remain mostly unknown. Previous studies have shown possible involvement of TGF-β in mouse ESCs while FasL has been implicated in rat ESC immune modulation [12], [13]. In our studies we treated ESC-derived factors with neutralizing antibodies against TGF-β in both T cell proliferation and in Foxp3 induction assays. We did not find a noticeable increase in T cell proliferation and a reduction in the number of Foxp3+ T cells in response to ESC-derived factors after neutralizing TGF-β (data not shown). Consistent with these results, Robertson et al. examined 87 immunologically relevant genes in mouse ESC and found that IL-10, TGF-β1, arginase-1, arginase-2 and indoleamine-2,3-dioxygenase did not contribute to their immune privilege [18]. Studies carried with human ESCs have shown that arginase-1 and HLA-G may play a role in ESC-mediated immune modulation [22], [41]. Based on these reports, it has become apparent that ESC-mediated immune modulation may occur through a number of different factors and mechanisms. Given the importance of ESCs in PKC-θ activation, our current focus has been to identify a specific factor that mediates such an effect.

### Conclusions

ESC-derived factors have the capacity to modulate T cell responses directly. In the presence of ESC-derived factors T cell proliferation is hindered and the expression of activation markers decreased. Moreover, ESC-derived factors favour a T regulatory phenotype over a Th1 response, leading to an increase in CD4+, CD25+ Foxp3+ cells and a decrease in IFN-γproduction. Such a modulation in T cell responses may be achieved by the ability of ESCs that specifically inhibit PKC-θ phosphorylation and its subsequent activity.

## Materials and Methods

### Cell lines and animals

Mouse ESC C57BL/6 cell line was obtained from ATCC. Mouse ESC D3 and J1 cells were kind gifts from Dr. Qiao Li and Dr. Michael Rudnicki, respectively (University of Ottawa, Ottawa, Ontario, Canada). Mouse strains C57BL/6, B6C3F1, Balb/c and CD1 (10 to 15 weeks old) were obtained from Charles River Laboratories, Montreal Canada. All hESC lines (H1 and H9 cell lines from WiCell Research Institute, Madison, WI, USA) [11], [14], [15], [20] were used with the approval of the local Ethics Board (Permit No. 2005721-01H) and the Stem Cell Oversight Committee of the Canadian Institutes for Health Research (Permit No. FRN-11224). Animals were maintained at the University of Ottawa (Ottawa Ontario, Canada) in accordance with the Canadian Council on Animal Care guidelines under protocols approved by the Animal Use Subcommittee at The University of Ottawa (Permit No. BMI-95).

### ESC conditioned medium

Mouse ESCs were grown in 75 cm^2^ Corning flasks (Sigma Inc.) in 10 ml of ESC medium for 24 hours. The next day the medium was harvested and centrifuged at 15000 g for 15 minutes at 4°C in order to remove cell debris. Supernatants were transferred to new tubes and either used right away in proliferation assays at indicated ratios in combination with RPMI or frozen at −20°C for later use. Mouse embryonic fibroblast (MEF) cells-conditioned medium as described above was used as a control.

### Mouse ESC extraction

Mouse ES lines D3, J1 and B6 were grown on mitomycin- treated MEF cells in Dulbecco's modified eagle Medium (DMEM) containing 4.0 mM L-glutamine, 1.0% non-essential amino acids, 0.10 µM 2-ME, 1.0×10^2^ units of Penicillin, 1.0×10^2^ units of Streptomycin and 15% FBS (Invitrogen Canada Inc., Burlington ON) supplemented with 1.0×10^3^ units/mL of LIF (Millipore Canada Ltd., Etobicoke ON) and incubated at 37°C with 5.0% CO_2_. Subsequently, the cells were cultured on 0.10% gelatin coated plates for two passages in order to eliminate MEF cells. Upon reaching confluence, the cells were harvested by treatment with trypsin (Invitrogen Inc.) and dissociated to obtain a single cell suspension. Subsequently, ESCs were washed twice with ice cold PBS and centrifuged at 400 g for 6 minutes at 4°C. After washing, the cells were re-suspended in lysis buffer. (50 mM HEPES, 50 mNaCl, 1.0 mM EDTA, 1.0 mM DTT, 50 mM L-arginine, pH 8.2). The lysis buffer was supplemented with pan protease inhibitors at 1∶100 (4-(2-aminoethyl) benzenesulfonyl fluoride (AEBSF), pepstatinA, E-64, bestatin, leupeptin, and aprotinin) dissolved in DMSO, Sigma Aldrich Canada Ltd, Oakvile ON). At this point the cells were incubated for 30 minutes on ice and sonicated until complete lysis of the cells was achieved. The sonicated cells were centrifuged at 15000 g for 15 minutes at 4°C to remove cell membrane, mitochondrial and nuclear fractions. The soluble cell free fraction was separated from the insoluble fraction and both stored at −80°C. Protein concentration was determined using Bio-Rad Protein assay kit (Bio-Rad Laboratories Ltd., Mississauga ON).

### Mouse splenocyte and CD3+ T cell isolation

Mouse spleens were removed aseptically and gently homogenized with the frosted ends of two sterile microscope slides and passed through a 45 µm mesh filter. The cells were washed twice with PBS and red blood cells were removed by Ficoll centrifugation or ACK red blood cell lysis buffer (Cederlane Laboratories Ltd. Burlington ON). Afterwards, cells were washed twice with PBS and re-suspended in media. Purified CD3+ T cells were obtained by negative selection using a magnetic labeling kit (StemCell Technologies Inc.) according to manufacturer instructions (Purity was 92% for CD3 marker).

### Mouse splenocyte activation

Isolated splenocytes were suspended in serum free RPMI media at 1.0×10^6^ cells/mL and stained with 0.01 µM of carboxyfluoresceindiacetatesuccinimidyl ester (CFSE) (Simga Aldrich Inc.) or CellTrace Violet Cell Proliferation kit (Invitrogen Inc.) for 40 minutes at 37°C. Subsequently, the cells were washed twice with PBS. Splenocytes were plated at 1.0×10^5^/well in 96 well plates in a total volume of 0.20 ml of RPMI media (10% FBS, 2 mM L-glutamine, 1×10^2^ U penicillin, 1×10^2^ U streptomycin, 1.0 mM Non-essential amino acids, 50 µM 2-mercapto-ethanol). Cells were stimulated with 1.2 µg/ml of anti-CD3 and 0.50 µg/ml of anti-CD28 (eBioscience Inc., San Diego CA) (unless otherwise stated) in the presence of increasing concentration of mESC-derived factors (0.15 mg, 0.23 mg/ml and 0.30 mg/ml). Splenocytes were also stimulated with either concanavalin A (ConA), or phorbol 12-myristate 13-acetate (PMA) and ionomycin in the presence or absence of 0.23 mg/ml of ESC-derived factors. Proliferation was allowed to proceed for 2–3 days and CFSE dilution was analyzed by Beckman Coulter Cyan flow cytometer.

### Mixed lymphocyte reaction

Splenocytes were isolated as described above. One-way MRL were carried out with 1.0×10^5^ splenocytes from both responder and stimulator cells in 96 well U bottom plates. Stimulator cells were pre-treated with 50 µg/mL of mitomycin C for 40 minutes at 37°C prior to MLR. The cells were allowed to proliferate for 3 days and tritium uptake was determined. The cells were harvested on to 96 well filters-mats (Wallac Inc., Turku Finland) using a TomTec harvester. Tritium uptake was determined by liquid scintillation using a Wallac 1450 Microbeta Plus liquid scintillation counter (Wallac Inc.). Results are displayed as counts per minute (CPM) of triplicate wells ± SD.

### T cell markers

T cell activation was examined using CD3, CD4, CD8, CD25, CD44 and CD69 (eBioscience Inc.). CD3+ T cells were stimulated with plate bound anti-CD3 and anti-CD28 (eBioscience Inc.) in the presence or absence of 0.23 mg/ml of ESC-derived factors and allowed to proliferate for the indicated times. Cells were harvested and washed with PBS. Next, blocking was carried out with 10% rat serum for 15 minutes on ice. At the end of the incubation period, antibodies were added to the cells according to manufacturer recommendation and the cells were incubated for 30 minutes. Cells were analyzed by Beckman Coulter Cyan flow cytometer. Data were analyzed by gating on CD3 positive cells followed by examination of activation markers CD25, CD44 and CD69 on CD4 and CD8 separately.

### Cell death assay

Splenocytes were stimulated with anti-CD3 and anti-CD28 (eBioscience Inc.) in the presence or absence of 0.23 mg/ml of ESC-derived factors and allowed to proliferate for 2–3 days in 96 well plates as described above. Cells were harvested and washed twice with PBS. At this point, cells were re-suspended in Annexin-V buffer and stained with 5.0 µl of Annexin V-PE for and anti-CD3-FITC antibody for 30 minutes (BD Biosciences Inc. Mississauga, ON). Cells also received 5 µl of 7-amino-actinomycin D (7AAD) for the last 10 minutes of incubation. Cell death was determined by flow cytometer using Beckman Coulter Cyan flow cytometer.

### QPCR

Splenocytes were isolated as described above. A one way mixed lymphocyte reaction was performed by treating stimulator cells with 50 µg/mL of mitomycin C for 40 minutes at 37°C prior to MLR. Subsequently, 1×10^6^ C57BL/6 splenocytes (responders) were incubated with 1×10^6^ CD1 splenocytes (stimulators) in 48 well plates in triplicates. The cells were treated with 0.30 mg/ml of ESC-derived factors or vehicle control. At the indicated time points cells were harvested, lysed and RNA was isolated using QiagenRNeasy Mini Kit (Qiagen Canada Inc. Mississauga ON) according to manufacturer instructions. Subsequently, cDNA was synthesized using QiagenQuantiTech Reverse Transcription kit (Qiagen) according to manufacturer instructions. QPCR was carried out with iQ SYBR Green Supermix (Bio-Rad Laboratories Ltd.) and My iQ-iCycler (Bio-Rad Laboratories Inc.) with an initial hot start for 90 seconds at 94°C followed by 40 cycles set for 10 seconds at 94°C, 30 seconds at 60°C, and 30 seconds at 72°C. Primers were as follows; IL-2 forward CAGGATGGAGAATTACAGGAACCT, IL-2 reverse 5′ TTTCAATTCTGTGGCCTGCTT, IFN-γ forward 5′ GAAAATCCTGCAGAGCCAGA, IFN-γ reverse 5′ TGAGCTCATTGAATGCTTGG, TGF-β forward 5′ GTGCTCGCTTTGTACAACAGC, TGF-β reverse 5′ TTACCAAGGTAACGCCAGG, Foxp3 forward 5′ CGAAAGTGGCAGAGAGGTATTGA, Foxp3 reverse 5′ ACTGTCTTCCAAGTCTCGTCTGAA, Tbet forward 5′ GCCAGGGAACCGCTTATATG and Tbet reverse 5′ GACGATCATCTGGGTCACATTGT. Gene expression levels were normalized to GAPDH and fold change was compared to relative gene expression with responder cells alone through the delta-deltaC_t_ method.

### Western blot

Two million splenocytes were-pretreated with 0.23 mg/ml of ESC-derived factors or vehicle control overnight in 0.50 ml of RPMI medium in 48 well plates. The next day, cells were stimulated with either 50 ng/ml of PMA or anti-CD3 and anti-CD28 for the indicated periods. Cells were harvested and lysed immediately in lysis buffer (25 mM Tris-HCl, 0.15 M NaCl, 5.0 mM MgCl_2_, 1.0% NP-40, 1.0 mM DTT, 5.0% glycerol, [pH 7.5]) and an equivalent volume of Laemmli Sample buffer (Bio-Rad Laboratories Ltd.) was added to the samples. Samples were boiled for 5 minutes and ran on a 10% SDS-PAGE gel and transferred to nitrocellulose membranes. The membranes were blocked with 5% powder milk (w/v) or 5% BSA (w/v) in TBS-T for 1 hour. Membranes were probed with rabbit anti-mouse phospho-PKC-θ (Thre538) and total PKC-θ antibodies at 1∶500 (Santa Cruz Biotechnology Inc. Santa Cruz, CA) overnight. Subsequently, membranes were washed 3 times with TBS-T for 15 minutes and were probed with goat anti-rabbit secondary HRP conjugated antibody. Again, membranes were washed 3 times with TBS-T. At this point, the bands were visualized with Amersham ECL Plus western blot detection systems (GE Healthcare Biosciences Corp. Piscataway, NJ). A similar procedure was carried out for pAKT (1∶3000), IκB-α (1∶500) and pPLC-γ (1∶1000) (Cell Signaling Technology Inc. Danvers, MA).

### Intracellular cytokine and transcription factor staining

Two million splenocytes were pre-treated with 0.23 mg/ml of ESC-derived factors or vehicle control overnight in 0.50 ml of RPMI media in 48 well plates. The next day, the cells were stimulated either with anti-CD3/CD28 or PMA and ionomycin for 6 hours. After the first 1-2 hours of stimulation cells were also treated with Protein Transport inhibitor cocktail (eBioscience Inc.). At the end of the incubation period cells were harvested and washed twice. Subsequently the cells were incubated with 10% rat serum and stained for surface markers CD4 and CD8. Staining was carried out for 30 minutes followed by two washes with PBS. The cells were fixed and permeabilized using the Foxp3 Fixation/Permeabilization Concentrate kit according to manufacturer's instructions (eBioscience Inc.). Cells were stained for intracellular IFN-γ and Foxp3 (eBioscience Inc.).

### Statistical analysis

Statistical significance was determined using a Student's *t-*test, ANOVA or chi-square wherever appropriate. Results were considered significant when *P*<0.05.

## Supporting Information

Figure S1 **A one-way MLR was performed with C57BL/6 splenocytes used as responder cells and BALB/c splenocytes used as stimulators.** Cells were treated with increasing concentration of ESC derived factors or equivalent volume of vehicle control. MLR was allowed to proceed for 4 days and tritiated thymidine was added for an additional 16–18 hours. Data points indicate counts per minute [CPM] of triplicate wells+/−SD. * indicates p<0.05.
(TIF)

Figure S2 **Purified human CD3+ T cells were stimulated with 30 ug/ml of ConA or 50 ng/ml of in the presence of hESC or vehicle control.** Tritiated thymidine was added on day 3 and the cells were cultured for an additional 16 to 18 hours. Results are displayed and counts per minute (CPM) of triplicate wells+/−SD.
(TIF)

Figure S3 **C57BL/6 splenocytes were stimulated with anit-CD3/anti-CD28 in the presence of ESC-derived factors or vehicle control for 72 hours.** The cells were harvested and washed with PBS and stained with anti-CD3-FITC, Annexin-V-PE and 7AAD to examine T cells apoptosis and necrosis. Analysis was carried out by gating on CD3+ cells.
(TIF)

Figure S4 **A one-way MLR was performed with C57BL/6 splenocytes used as responder cells and BALB/c splenocytes used as stimulators.** Cells were treated with 0.225 mg/ml of ESC derived factors or equivalent volume of vehicle control at start of culture. a) CD8 cells were examined for intracellular IFN-γ 24 hours later. b) Intracellular Foxp3 was examined 48 hours following start of culture in CD4+ CD25+ cells.
(TIF)

Figure S5 **Purified C57BL/6 T cells were pre-treated overnight with 0.23 mg/ml of ESC derived factors or equivalent volume of vehicle control.** The next day cells were stimulated with 50 ng/ml of PMA for the indicated amount of times. Cells were harvested and total nuclear protein was isolated. Samples were examined for translocated NFκB in each sample. Histone deacetylase 1 (HDAC1) was used as a loading control.
(TIF)
