# Supplementary figures and images for: Embryonic Stem Cell-Derived Factors Inhibit T Effector Activation and Induce T Regulatory Cells by Suppressing PKC-θ Activation
Source: PLoS One. 2012 Mar 7;7(3):e32420. doi: 10.1371/journal.pone.0032420 (PMC3296708; doi:10.1371/journal.pone.0032420)

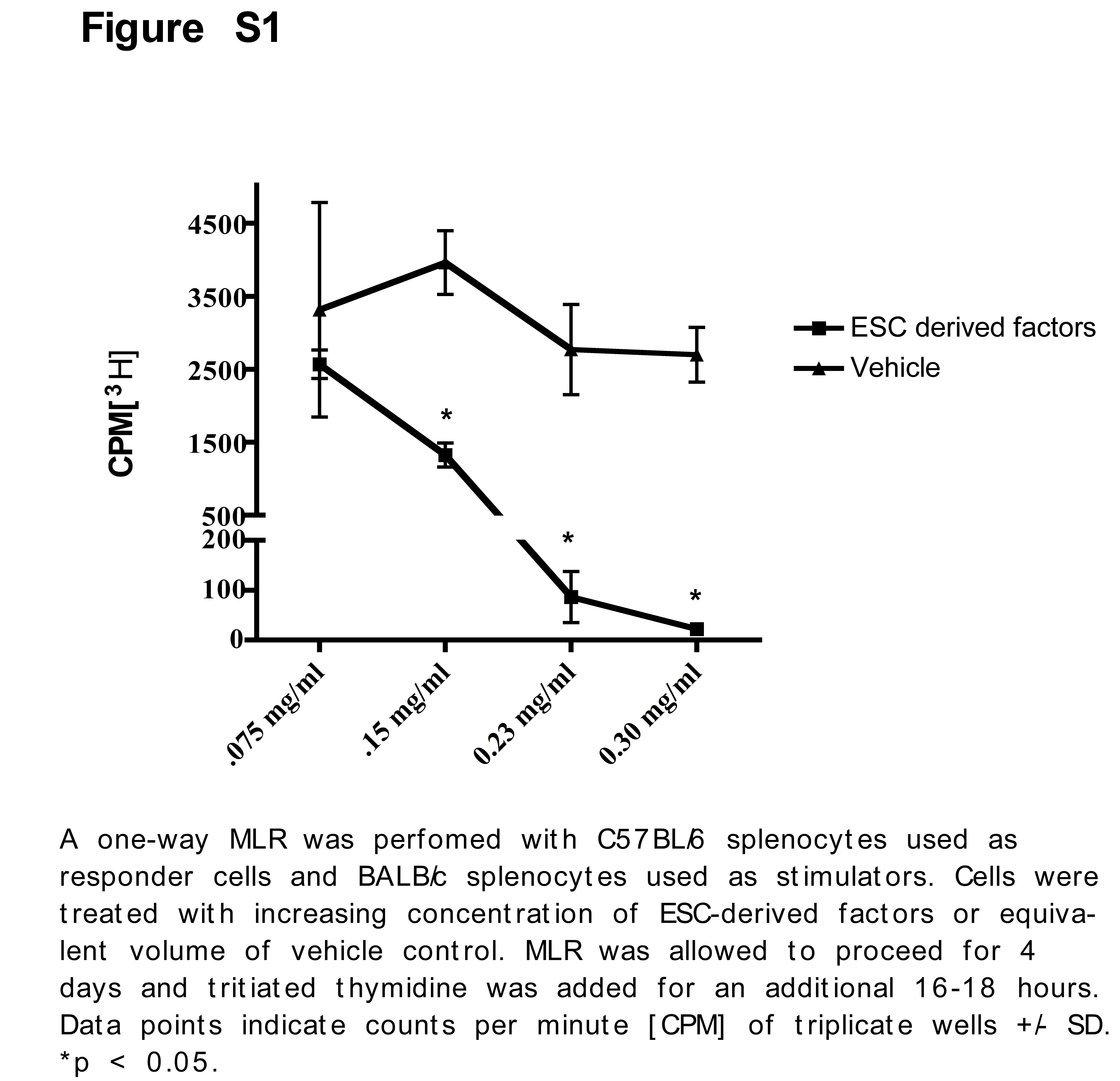

Supplement: Figure S1 — A one-way MLR was performed with C57BL/6 splenocytes used as responder cells and BALB/c splenocytes used as stimulators. Cells were treated with increasing concentration of ESC derived factors or equivalent volume of vehicle control. MLR was allowed to proceed for 4 days and tritiated thymidine was added for an additional 16–18 hours. Data points indicate counts per minute [CPM] of triplicate wells+/−SD. * indicates p<0.05. (TIF) [file pone.0032420.s001.tif]

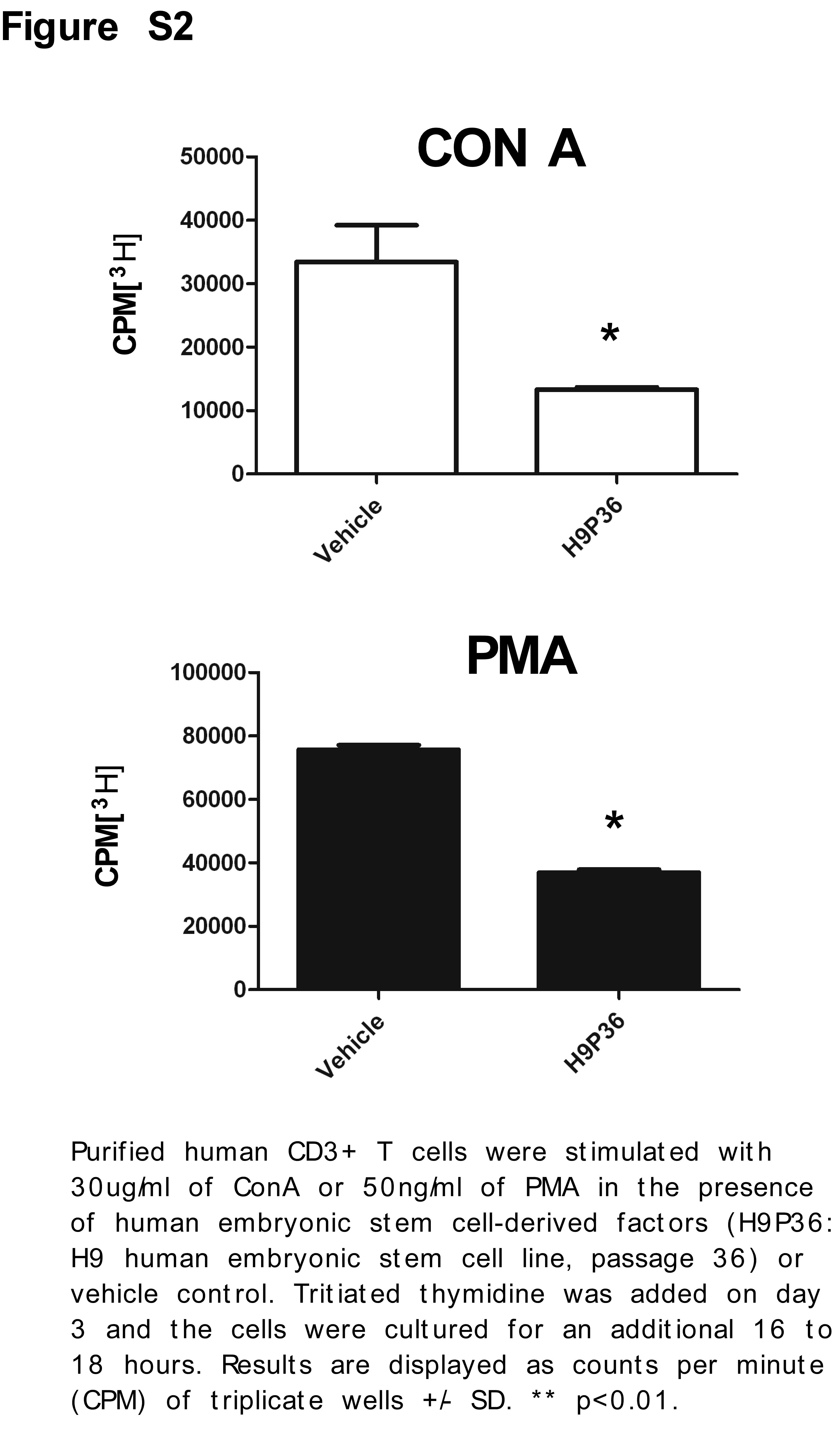

Supplement: Figure S2 — Purified human CD3+ T cells were stimulated with 30 ug/ml of ConA or 50 ng/ml of in the presence of hESC or vehicle control. Tritiated thymidine was added on day 3 and the cells were cultured for an additional 16 to 18 hours. Results are displayed and counts per minute (CPM) of triplicate wells+/−SD. (TIF) [file pone.0032420.s002.tif]

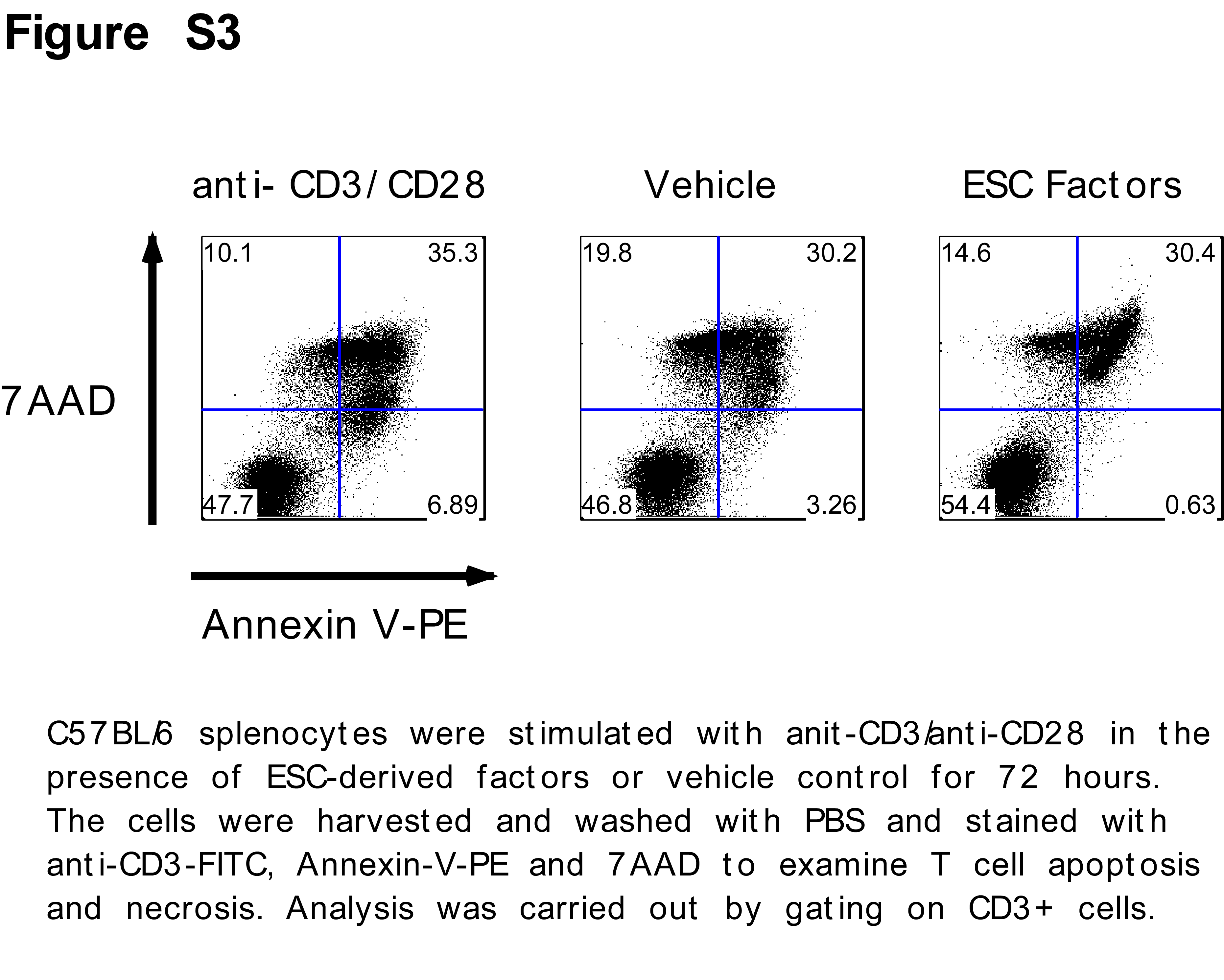

Supplement: Figure S3 — C57BL/6 splenocytes were stimulated with anit-CD3/anti-CD28 in the presence of ESC-derived factors or vehicle control for 72 hours. The cells were harvested and washed with PBS and stained with anti-CD3-FITC, Annexin-V-PE and 7AAD to examine T cells apoptosis and necrosis. Analysis was carried out by gating on CD3+ cells. (TIF) [file pone.0032420.s003.tif]

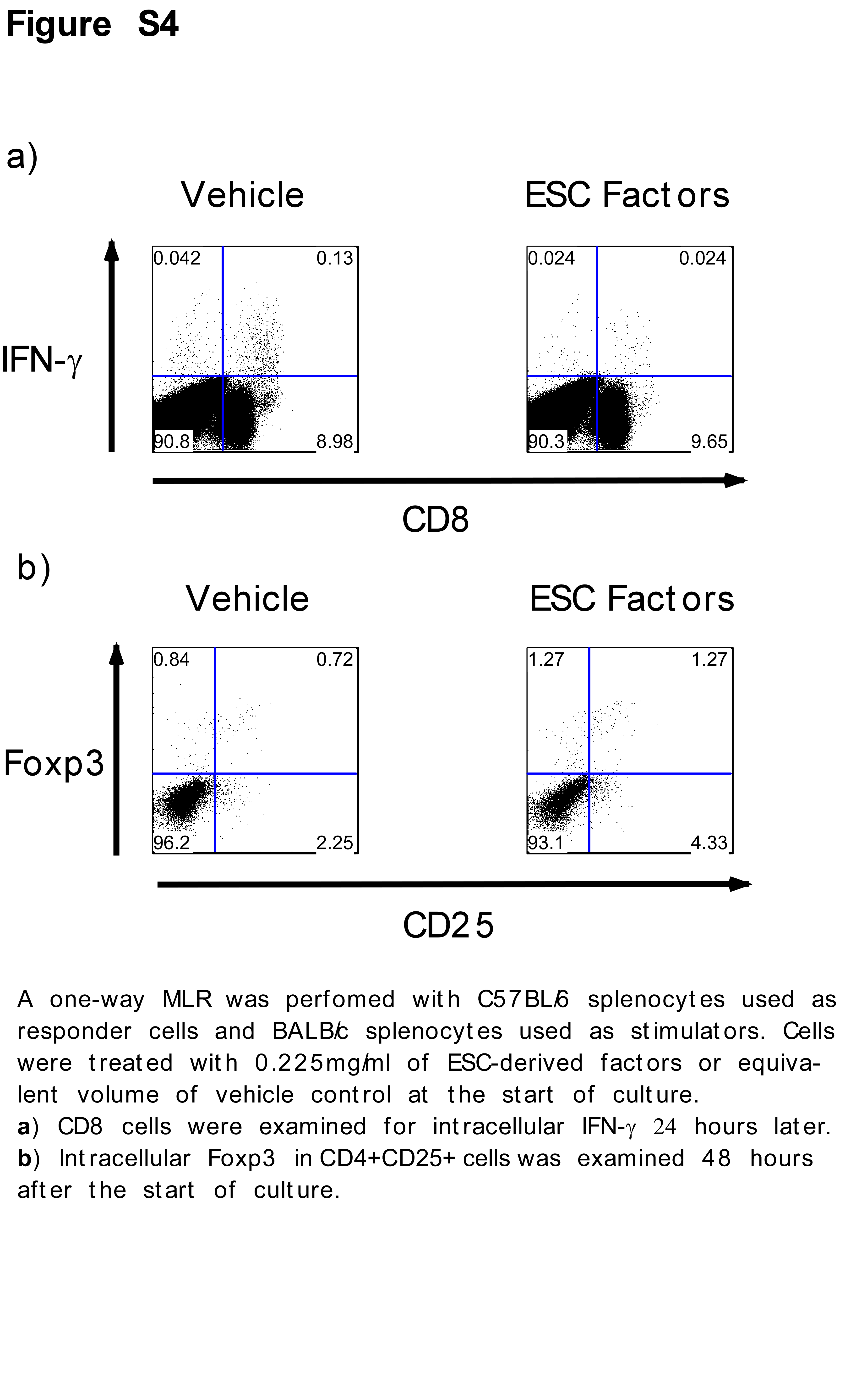

Supplement: Figure S4 — A one-way MLR was performed with C57BL/6 splenocytes used as responder cells and BALB/c splenocytes used as stimulators. Cells were treated with 0.225 mg/ml of ESC derived factors or equivalent volume of vehicle control at start of culture. a) CD8 cells were examined for intracellular IFN-γ 24 hours later. b) Intracellular Foxp3 was examined 48 hours following start of culture in CD4+ CD25+ cells. (TIF) [file pone.0032420.s004.tif]

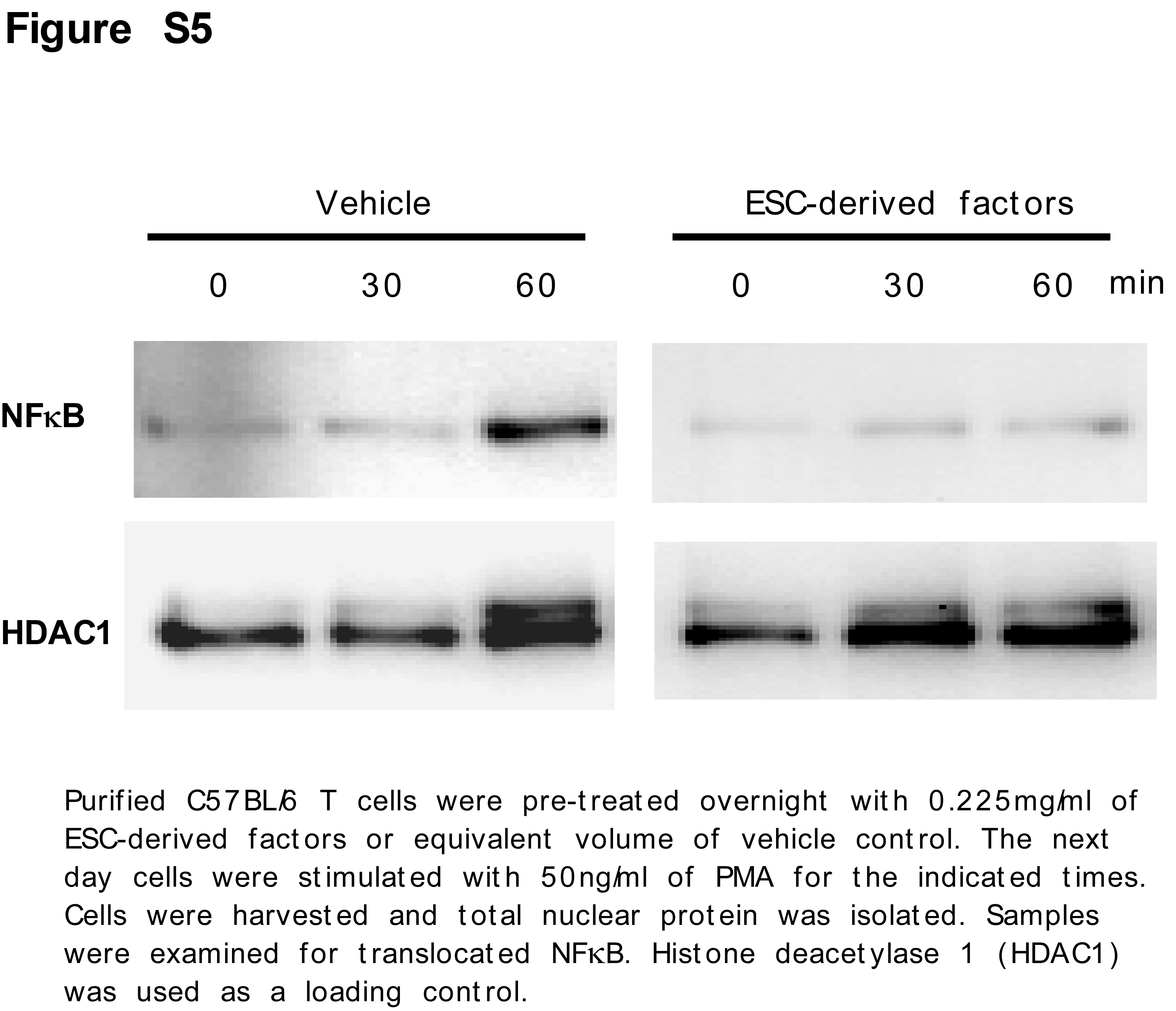

Supplement: Figure S5 — Purified C57BL/6 T cells were pre-treated overnight with 0.23 mg/ml of ESC derived factors or equivalent volume of vehicle control. The next day cells were stimulated with 50 ng/ml of PMA for the indicated amount of times. Cells were harvested and total nuclear protein was isolated. Samples were examined for translocated NFκB in each sample. Histone deacetylase 1 (HDAC1) was used as a loading control. (TIF) [file pone.0032420.s005.tif]
